# Apical Sparing in Routine Echocardiography: Occurrence and Clinical Significance

**DOI:** 10.3390/jcdd11090262

**Published:** 2024-08-27

**Authors:** Marina Leitman, Vladimir Tyomkin

**Affiliations:** 1 Department of Cardiology, Shamir Medical Center, Zerifin 70300, Israel; 2 Sackler School of Medicine, Tel Aviv University, Tel Aviv 6997801, Israel

**Keywords:** apical sparing, amyloidosis, hypertrophic cardiomyopathy, aortic stenosis, end-stage renal disease, hypertension

## Abstract

Apical sparing is an echocardiographic pattern where myocardial strain is preserved at the apex compared to the basal segments. In a normal heart, longitudinal strain shows a gradient with lower values at the base and higher at the apex. This gradient becomes more pronounced in pathological states, such as cardiac amyloidosis, resulting in a relative apical sparing effect. This study explores cardiac conditions associated with apical sparing and the underlying mechanisms. We reviewed echocardiography examinations reporting apical sparing from 2021 to 2024 in our hospital database. Relevant echo exams and clinical data were retrieved and analyzed. Apical sparing was identified in 74 patients. Cardiac amyloidosis was diagnosed in 12 patients (16.2%). Other cardiac pathologies potentially contributing to apical sparing included hypertrophic cardiomyopathy, left ventricular hypertrophy due to hypertension, end-stage renal disease, coronary artery disease (involving the right coronary artery and left circumflex), reversed Takotsubo syndrome, and chemotherapy-induced cardiomyopathy. The clinical context of echocardiography was crucial in guiding the diagnostic work-up. Apical sparing is a nonspecific echocardiographic finding associated with various cardiac conditions. Its diagnostic value depends heavily on the clinical context. Understanding the broader clinical picture is essential for accurate interpretation and diagnosis.

## 1. Introduction

Apical sparing is a phenomenon frequently observed in echocardiography, characterized by a distinctive pattern where the apex of the heart shows relatively preserved myocardial strain compared to the basal segments. In a normal heart, longitudinal strain is typically heterogeneous, with the lowest values at the base and the highest at the apex [1]. This gradient can become especially pronounced in certain pathological conditions, resulting in a relative apical sparing effect. 

Cardiac amyloidosis, characterized by the deposition of amyloid proteins within the myocardial tissue, is a condition where apical sparing has garnered significant attention. In the context of suspected cardiac amyloidosis, the presence of apical sparing can be a crucial echocardiographic finding that aids in diagnosis. A recent large study [2] identified several multivariate predictors of cardiac amyloidosis in patients with apical sparing, including age, visual pattern of apical sparing, the ratio of left ventricular ejection fraction to global longitudinal strain (>4.1), and an apical sparing ratio ≥1, with apical sparing defined as the ratio of average apical strain to the sum of average basal and mid-segment strain. The study found that cardiac amyloidosis was less likely in patients with coronary artery disease and hypertension, although hypertension was frequently associated with amyloidosis (34%). Importantly, apical sparing is not exclusive to cardiac amyloidosis and can be observed in various other cardiac conditions associated with hypertrophy. For instance, a recent prospective study [3] investigated the myocardial work index, which combines longitudinal strain and systolic blood pressure. The study revealed that myocardial work parameters, in particular global work index and global constructive work, were significantly lower in patients with transthyretin amyloidosis compared to those with hypertrophic cardiomyopathy and hypertensive heart disease. Although apical sparing was not specifically reported in this study, myocardial work metrics offer valuable insights for evaluating patients with cardiac hypertrophy and distinguishing between different pathologies.

Out of the 30 known misfolded amyloid proteins, only nine accumulate in the heart [4]. The most prevalent is amyloid light chain (AL) amyloidosis, often associated with blood cell dyscrasias and affecting the heart in approximately 70% of cases. The second most common type is transthyretin amyloidosis (ATTR), involving the extracellular deposition of transthyretin tetramers produced in the liver. ATTR can be categorized into wild-type (ATTRwt) and variant forms (ATTRv). ATTRwt affects the heart in nearly 100% of cases, while ATTRv involves the heart in 30–100% of cases, depending on the specific variant. Amyloid deposits commonly accumulate in cardiac valves and the myocardium, predominantly impacting older patients, especially women. In the elderly, lipid accumulation can lead to inflammation and endothelial damage, resulting in fibrosis, calcification, and stenosis of the aortic valve. In ATTR amyloidosis, transthyretin tetramers infiltrate the aortic valve leaflets, worsening aortic stenosis and often leading to low-flow, low-gradient aortic stenosis, particularly in males. The extracellular deposition of amyloid is associated with collagen accumulation and fibrosis, progressively impairing myocyte function and leading to heart failure and arrhythmias. Given these complexities, a high level of clinical suspicion is essential for diagnosis. While echocardiography and the presence of extracardiac red flags can raise clinical suspicion, a definitive diagnosis typically requires multimodal imaging and/or biopsy.

Cardiac magnetic resonance (CMR) is the most effective noninvasive method for myocardial tissue characterization [4]. Although late gadolinium enhancement may be absent in the early stages of amyloidosis, T1 and T2 parametric mapping, as well as extracellular volume (ECV) mapping, are valuable for detecting cardiac amyloidosis. Elevated native T1 and ECV values are indicative of amyloidosis. However, distinguishing between different types of amyloidosis often requires additional evaluation. For instance, protein P, which stabilizes amyloid fibrils, exhibits a high calcium affinity in ATTR amyloidosis. Therefore, scintigraphy with Tc-99m-PYP is crucial for the noninvasive diagnosis and grading of ATTR amyloidosis [4]. When ATTR amyloidosis is strongly suspected based on clinical and echocardiographic findings, a Tc-99m-PYP scan is typically sufficient for noninvasive diagnosis. Nonetheless, a comprehensive echocardiographic examination remains essential for the diagnosis of cardiac amyloidosis.

In this work, we aim to explore the diverse cardiac conditions associated with apical sparing and provide insights into the underlying mechanisms. We will discuss why apical sparing is not universally present in all cases of cardiac amyloidosis during echocardiographic examinations, thereby highlighting the complexity of this echocardiographic feature. By examining various clinical scenarios and the associated echocardiographic findings, we hope to elucidate the diagnostic implications and limitations of apical sparing, emphasizing its role within the broader context of clinical practice.

## 2. Materials and Methods

We examined the hospital database for echocardiography examinations from 2021 to 2024 where apical sparing was reported. All studies were retrieved and analyzed, along with the clinical data of these patients, using digital hospital files. We analyzed clinical conditions that could potentially cause apical sparing. Echocardiography examinations were reviewed according to the latest guidelines on cardiac amyloidosis. All parameters included in the amyloidosis multiparametric score were calculated. The apical-to-basal strain ratio was determined as recommended in the recent guidelines, using the septal apical-to-basal ratio [5,6].

### 2.1. Inclusion Criteria

Patients with echocardiography reports from 2021 to 2024 in which apical sparing was documented as part of the strain analysis.

Cases with clinical and echocardiographic data available in digital hospital records for review.

### 2.2. Exclusion Criteria

Patients for whom apical sparing was not confirmed upon manual review of echocardiography studies.

Echocardiography studies with incomplete or poor-quality strain data preventing accurate calculation of apical-to-basal strain ratio.

Patients with missing clinical data critical for the analysis.

#### Echocardiographic Assessment

The echocardiographic assessment was performed according to current guidelines. All studies were conducted using the VIVID E95 v203 (GE Healthcare, Horten, Norway) echocardiography machine, with digital storage of all examinations. Standard assessments included linear and volumetric measurements of cardiac chambers and an evaluation of diastolic function. Diastolic function parameters included mitral inflow velocities (E and A waves), E-wave deceleration time, E/A ratio, and tissue Doppler imaging of the mitral annular septal velocities (s’ and e’). Additionally, global longitudinal strain (GLS) was calculated in real time for each examination, using speckle-tracking echocardiography imaging.

### 2.3. Sample Size Calculation

The sample size for this study was determined retrospectively based on echocardiography exams conducted between 2021 and 2024. Prior research, such as the study by Phelan et al. [7], evaluated the diagnostic accuracy of apical sparing in cardiac amyloidosis, including 55 patients with amyloidosis and 30 controls. Given our aim to investigate a broader range of conditions associated with apical sparing, our sample size of 74 patients is comparable and considered sufficient to achieve statistical power for detecting differences in strain patterns across varied cardiac pathologies.

### 2.4. Statistical Methods

Descriptive statistics were computed to summarize each parameter’s characteristics at each time point. Continuous data were presented as means ± standard deviations. The normal distribution of all differences was assessed using the Kolmogorov–Smirnov test. A two-tailed, dependent *t*-test was utilized for continuous variables. Categorical data were reported as numbers and percentages. Univariate analysis was conducted using the chi-square or Fisher’s exact test, as appropriate, to determine significant variables (*p* < 0.05). The statistical analysis was conducted using IBM SPSS Statistics for Windows, Version 28.0 (Armonk, NY, USA: IBM Corp).

### 2.5. Ethical Approval

Formal approval by the Ethics (Helsinki) Committee at Shamir (Assaf Harofeh) Medical Center was not required for this retrospective study. All methods were carried out following relevant guidelines and regulations. 

## 3. Results

**Figure 1:**
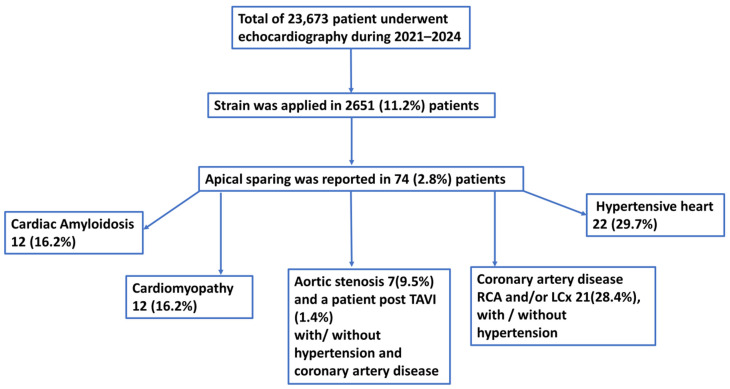
Flow chart of patients in whom apical sparing was reported in routine echocardiography examinations. Strain assessment was conducted in 11.2% of patients; of them apical sparing was reported in 74 patients (2.8%). Cardiac amyloidosis, cardiomyopathies, aortic stenosis, coronary artery disease with involvement of right and/or left circumflex coronary artery, hypertension, and end-stage renal disease were found as a presumed mechanism of apical sparing. TAVI—transcutaneous valve implantation, HPTN—hypertension, CAD—coronary artery disease, RCA—right coronary artery, LCx—left circumflex coronary artery.

Between 2021 and 2024, echocardiography examinations were performed on 23,673 patients (see Figure 1). Among these, strain analysis was conducted on 2651 patients (11.2%). Within this group, we identified 74 patients for whom apical sparing was reported during echocardiography examinations. Strain assessment at our hospital is typically performed on patients undergoing chemotherapy and those with left ventricular hypertrophy. All patients were referred for echocardiography by the internal medicine departments and underwent work-up based on the clinical picture, as decided by the responsible physicians.

The general characteristics of the patients and echocardiography data are summarized in Table 1 and Figure 1. The mean age was 71.2 ± 14.2 years, with 48 males (64.9%) and 26 females (35.1%). Cardiac amyloidosis was confirmed in 12 patients (16.2%): 6 with transthyretin (TTR) amyloidosis, 5 with light-chain (AL) amyloidosis, and 1 with renal amyloidosis.

Among the other patients with apical sparing, 36 (37.8%) had hypertension, and 21 (28.4%) had coronary artery disease affecting the right coronary artery and left circumflex coronary artery. Of these, six patients had undergone coronary artery bypass grafting (CABG) with a patent left internal mammary artery (LIMA) to the left anterior descending artery (LAD). Seven patients had significant aortic stenosis, and one had undergone transcatheter aortic valve implantation (TAVI). Twelve patients had cardiomyopathy: three had hypertrophic cardiomyopathy, three had left ventricular nondilated cardiomyopathy, three had cardiomyopathy post-chemotherapy, and one had transient cardiomyopathy during sepsis. Additionally, two patients suffered from reversed Takotsubo cardiomyopathy.

In 10 hypertensive patients, coronary artery disease involving the right and left circumflex coronary arteries was reported. Three patients with significant aortic stenosis and one patient post-TAVI were also hypertensive. One patient with aortic stenosis also had coronary artery disease. Additionally, one post-chemotherapy patient had both aortic stenosis and nondilated left ventricular cardiomyopathy.

End-stage renal disease was detected in 14 patients (18.9%); in 7 of these cases, it was associated with hypertension, and in 3 cases, it was associated with both hypertension and coronary artery disease.

During echocardiography examinations, most patients were in sinus rhythm (57 patients, 77%). The mean ejection fraction was mildly reduced at 47.9% ± 9.7%. Global longitudinal strain was moderately reduced at −11.5% ± 2.6%. The mean apical/basal strain ratio was 5.1 ± 4.5. 

Regional wall motion abnormalities, predominantly in the basal infero-posterior segments, occurred in 19 patients (25.7%). The left ventricular mass index and relative wall thickness were increased, consistent with concentric remodeling. Grade II diastolic dysfunction, indicated by an E/E’ ratio of 16.8 ± 6.3, suggested increased left ventricular filling pressure.

Echocardiography data for patients with cardiac amyloidosis were compared with data from other patients with apical sparing (Table 2). Regional wall motion abnormalities were less common in patients with cardiac amyloidosis (group 1) than in other patients with apical sparing (group 2), occurring in 2 (16.7%) vs. 17 (27.4%) patients, respectively (*p* = 0.0006). Patients with amyloidosis had more pronounced left ventricular hypertrophy, with a left ventricular mass index of 167.8 ± 31.6 compared to 143.4 ± 46.1 (*p* = 0.04), largely due to a thicker posterior wall (1.4 ± 0.23 vs. 1.2 ± 0.29, *p* = 0.019). 

Left ventricular filling pressure was also higher in patients with cardiac amyloidosis, as indicated by an E/E’ ratio of 22 ± 6.4 compared to 15.8 ± 5.7 (*p* < 0.02).

**Figure 2:**
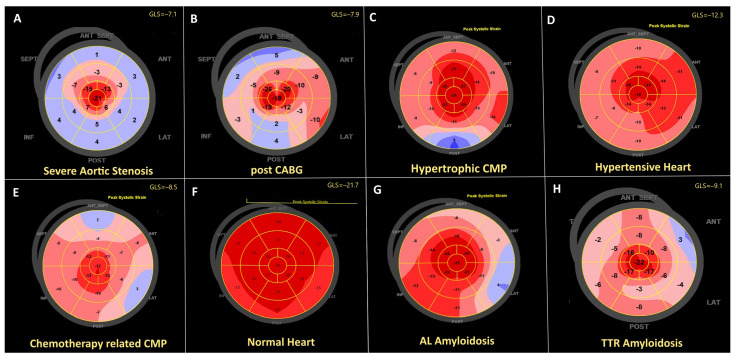
Example of peak longitudinal systolic strain distribution over the left ventricle in different clinical scenarios. (**A**)—72 years female with severe aortic stenosis. (**B**)—74 years hypertensive patient post CABG and patent LIMA to LAD, basal wall motion abnormalities attributed to the ischemic heart diseases are associated with reduced basal strain. (**C**)—40 years male with hypertrophic cardiomyopathy. (**D**)—42 years patient with hypertension. (**E**)—71 years female with chemotherapy related cardiomyopathy, reduced global strain with a relative apical sparing. (**F**)—Healthy 31 years old male. Physiological apical sparing is seen. (**G**)—68 years patient with AL amyloidosis. (**H**)—81 years patient with ATTR amyloidosis.

Examples of bull’s-eye plots of longitudinal strain with apical sparing related to different cardiac pathologies are shown in Figure 2.

## 4. Discussion

In our study of 74 patients, we observed that various cardiac pathologies contribute to apical sparing. Cardiac amyloidosis was confirmed in 16.2% of the patients. Other conditions associated with apical sparing included hypertension, either alone or in conjunction with coronary artery disease and aortic stenosis. End-stage renal disease (ESRD), whether alone or combined with hypertension and/or coronary artery disease, was frequently observed in patients with apical sparing. We also noted coronary artery disease involving the right and left circumflex coronary arteries, including cases with a patent graft to the left anterior descending coronary artery following coronary artery bypass grafting. Various types of cardiomyopathies, such as hypertrophic cardiomyopathy, nondilated left ventricular cardiomyopathy (as recognized in recent guidelines [8]), reversed Takotsubo syndrome, and post-chemotherapy cardiomyopathy, can also exhibit the characteristic apical sparing pattern.

In patients with amyloidosis, hypertrophy was more pronounced, as evidenced by significantly higher left ventricular mass and posterior wall thickness. The E/E’ ratio was significantly higher in patients with cardiac amyloidosis compared to those without. However, the apical-to-basal strain ratio and global longitudinal strain did not differ significantly between those with and without amyloidosis.

According to the previous literature, “apical sparing” is both specific and sensitive, with a sensitivity of 93% and a specificity of 82%, when compared to controls and patients with other forms of left ventricular wall thickening [9]. Studies have shown that the basal-to-apical gradient reflects the amyloid burden in all types of cardiac amyloidosis, as detected by MRI and histopathology. Reduced apical strain has been associated with major adverse cardiovascular events (MACEs) and advanced New York Heart Association (NYHA) functional class III to IV heart failure [9]. However, a recent echocardiography–histopathology study presented a different perspective, identifying three types of amyloid deposition: (1) diffuse low-burden amyloid deposition with mildly reduced or normal strain, (2) predominantly basal amyloid deposition with relative apical sparing on echocardiography, and (3) diffuse high-burden amyloid deposition with severely reduced strain. These findings suggest that these represent stages of cardiac amyloid deposition rather than distinct types [10].

The multiparametric score for echocardiographic detection of cardiac amyloidosis assesses its diagnostic value in patients with unexplained left ventricular wall thickness ≥ 12 mm. This score was initially proposed for detecting AL amyloidosis [11], where unexplained left ventricular hypertrophy is relatively uncommon. In wild-type ATTR amyloidosis, the term “unexplained” left ventricular hypertrophy is more ambiguous, as many elderly patients with ATTR amyloidosis may have hypertension or aortic stenosis, complicating the diagnosis. 

In addition to predominant amyloid deposition in the basal cardiac segments, increased shear stress in these segments has also been proposed as a mechanism for apical sparing [12]. A higher degree of apoptosis and remodeling at the base of the heart forms the pathophysiological substrate for apical sparing [13]. However, apical sparing alone cannot distinguish between different forms of cardiac amyloidosis [14]. Multiple conventional and advanced speckle tracking imaging parameters and indexes have been suggested for diagnosing amyloidosis, including multidimensional strain calculations. The ejection fraction to global longitudinal strain ratio has been identified as the best discriminator of cardiac amyloidosis, even in patients with normal ejection fractions and mild to moderate hypertrophy.

Diastolic function is significantly impaired in cardiac amyloidosis, with high filling pressure reflected by impaired left atrial function [15]. Significant impairment of left atrial strain has been observed in cardiac amyloidosis compared to hypertrophic cardiomyopathy, even in patients with preserved ejection fraction.

Later studies have shown that apical sparing is not exclusive to amyloidosis [16]. For example, relative apical sparing has been observed in patients with severe aortic stenosis without amyloidosis, correlating with left ventricular hypertrophy [17]. In these patients, increased wall stress in the basal segments, along with predominant basal myocardial fibrosis and frequently observed mitral ring calcification, may contribute to the observed apical sparing. 

Patients with hypertension often exhibit apical sparing [18,19], as do those with various types of hypertrophy, including hypertrophic cardiomyopathy [20]. Recent studies have also highlighted an association between end-stage renal disease (ESRD) and apical sparing strain patterns [21,22,23]. This observation aligns with our findings, where ESRD, frequently associated with hypertension, contributes to left ventricular hypertrophy (LVH) and subsequently influences strain patterns, including apical sparing. 

The pathophysiological basis of apical sparing can be understood through the unique architecture of the normal heart. Research indicates that left ventricular strain varies across the myocardium, exhibiting both circumferential and longitudinal inhomogeneity [1]. In healthy individuals, longitudinal and circumferential strains are most pronounced in the endocardium and diminish towards the epicardium. Longitudinal strain shows a base-to-apex gradient, increasing towards the apex, while epicardial longitudinal strain remains relatively uniform. Circumferential strain, generally higher than longitudinal strain, also follows a gradient, being lower at the base and higher at the apex. These strain patterns are influenced by myocardial fibers orientation. While apical strain is typically the highest in a normal heart, conditions such as hypertrophic cardiomyopathy can accentuate the base-to-apex gradient due to increased shear stress, myocyte hypertrophy, apoptosis, and replacement fibrosis. In cardiac amyloidosis, amyloid deposition, along with shear stress and reduced myocardial function, contributes to apical sparing. Similarly, in coronary artery disease with basal segment involvement, apical sparing can be exaggerated. Accurate diagnosis and management of these conditions critically depend on understanding the clinical context and the specific strain patterns observed.

### 4.1. Impact on Future Management

Identifying apical sparing can guide clinicians toward specific diagnoses, such as cardiac amyloidosis, prompting the use of advanced imaging like CMR and Tc-99m-PYP scintigraphy for accurate diagnosis, which is crucial for initiating appropriate treatment. Apical sparing highlights the need to consider a range of conditions, leading to more comprehensive evaluations and reducing the risk of misdiagnosis. Recognizing apical sparing can influence treatment strategies by emphasizing the need for targeted interventions, particularly in cases of cardiac amyloidosis or left ventricular hypertrophy to mitigate cardiac damage. Understanding apical sparing helps tailor management strategies based on individual patient profiles, taking into account clinical context, patients’ age, and comorbidities. Further research is needed to validate the diagnostic value of apical sparing and explore its role in predicting disease progression and treatment response. Finally, the identification of apical sparing can enhance diagnostic accuracy, refine clinical pathways, guide treatment, and support personalized care, ultimately improving patient management.

### 4.2. Gaps in Evidence and Future Directions

Despite the insights gained from this study, several gaps in evidence remain. First, current studies, including ours, highlight the prevalence of apical sparing but do not fully elucidate its role in distinguishing between different types of cardiac pathologies, particularly in the context of overlapping clinical presentations.

Second, although advanced imaging techniques like CMR and Tc-99m-PYP scintigraphy are used for diagnosing cardiac amyloidosis, their integration with echocardiographic findings in a comprehensive diagnostic approach is still under exploration. Future research should focus on defining how echocardiographic patterns, including apical sparing, can be best utilized alongside these advanced modalities to enhance diagnostic precision.

Third, the pathophysiological mechanisms underlying apical sparing in conditions beyond cardiac amyloidosis, such as hypertension, ESRD, and various cardiomyopathies, need further investigation. Understanding these mechanisms could provide deeper insights into how apical sparing reflects disease severity and progression.

Additionally, longitudinal studies are needed to assess the prognostic value of apical sparing. While current evidence suggests an association with adverse outcomes, more robust data are required to determine how apical sparing might predict disease progression and response to treatment over time. 

Addressing these gaps will be crucial for refining the diagnostic and prognostic utility of apical sparing, ultimately leading to better patient management and tailored treatment strategies.

## 5. Conclusions

In summary, apical sparing observed in routine echocardiography is a nonspecific finding that can be associated with a variety of cardiac conditions. While it has been notably linked with cardiac amyloidosis, our study reveals that this pattern is also present in patients with diverse cardiac pathologies such as hypertension, coronary artery disease, aortic stenosis, and end-stage renal disease, among others. The diagnostic value of apical sparing is highly context-dependent, requiring careful consideration of the clinical setting and other echocardiographic findings.

## Figures and Tables

**Table 1:** General and echocardiography patients’ characteristics.

Variable	Value
Patients’ number	74
Age, years	71.2 ± 14.2
Female n (%)	26 (35.1%)
Male n (%)	48 (64.9%)
RCA and/or LCX CAD	21 (28.4%)
Cardiac amyloidosis n (%)	12 (16.2%)
Cardiomyopathies n (%)	12 (16.2%)
Hypertension n (%)	36 (48.6%)
Aortic stenosis n (%)	7 (9.5%)
s/p TAVI n (%)	1 (1.4%)
End-stage renal disease n (%)	14 (18.9%)
Body surface area, m^2^	1.8 ± 0.21
Sinus rhythm n (%)	57 (77%)
Atrial fibrillation n (%)	17 (23%)
Ejection fraction, %	47.9 ± 9.7
RWMA n (%)	19 (25.7)
IVS thickness, cm	1.5 ± 0.3
PW thickness, cm	1.2 ± 0.3
LVEDD, cm	4.8 ± 0.7
Relative wall thickness	0.59 ± 0.17
LVMi, g/m^2^	147.4 ± 45
Global longitudinal strain, %	−11.5 ± 2.6
Apical/basal strain ratio	5.1 ± 4.5
E/E’ ratio	16.8 ± 6.3
Maximal diastolic E’ velocity, cm/s	6.0 ± 2.0
Diastolic dysfunction grade	2.3 ± 0.7
PAP, mm Hg	40.8 ± 13.9
TAPSE, cm	1.9 ± 0.4

RCA—right coronary artery, LCX—left circumflex coronary artery, CAD—coronary artery disease, TAVI—transcutaneous aortic valve implantation, RWMA—regional wall motion abnormalities, IVS—interventricular septum, PW—posterior wall, LVEDD—left ventricular end diastolic diameter, LVMi—left ventricular mass index, PAP—pulmonary artery pressure, TAPSE—tricuspid annulus peak systolic exertion.

**Table 2:** Comparison of echocardiography data of patients with amyloidosis and other patients with apical sparing.

Variable	Group 1	Group 2	*p*-Value
Patients’ number	12	62	NA
Sinus n (%)	9 (75%)	48 (77.4%)	0.68
Ejection fraction, %	48.2 ± 7.9	47.8 ± 10.0	0.89
RWMA n (%)	2 (16.7%)	17 (27.4%)	0.0006
Global longitudinal strain, %	−10.4 ± 2.2	−11.6 ± 2.8	0.12
Apical/basal strain ratio	6.6 ± 5.0	4.9 ± 4.4	0.3
IVS thickness, cm	1.66 ± 0.15	17 (27.4%)	0.53
PW thickness, cm	1.4 ± 0.23	1.2 ± 0.29	0.019
LVEDD, cm	4.78 ± 0.58	4.81 ± 0.74	0.86
RWT	0.66 ± 0.13	0.57 ± 0.18	0.08
LWMi, g/m^2^	167.8 ± 31.6	143.4 ± 46.1	0.04
E/E’ ratio	22 ± 6.4	15.8 ± 5.7	*p* < 0.02
TAPSE, cm	1.85 ± 0.34	1.95 ± 0.37	0.38
PAP, mm Hg	45 ± 8.9	39.9 ± 14.6	0.14
Max E velocity, cm/s	5.6 ± 1.8	6.1 ± 2.1	0.45
Diastolic dysfunction grade	2.5 ± 0.5	2.2 ± 0.78	0.25

Group 1—patients with cardiac amyloidosis, Group 2—other patients with apical sparing, RWMA—regional wall motion abnormalities, IVS—interventricular septum, PW—posterior wall, LVEDD—left ventricular diastolic diameter, RWT—relative wall thickness, LVMi—left ventricular mass index, TAPSE—tricuspid annulus peak systolic exertion.

## Data Availability

The data presented in this study are available from the corresponding author upon reasonable request.
